# Supplementary material for: WordCommentsAnalyzer: A windows software tool for qualitative research
Source: F1000Res. 2018 Sep 4;7:536. Originally published 2018 May 3. [Version 2] doi: 10.12688/f1000research.14819.2 (PMC6137411; doi:10.12688/f1000research.14819.2)
Supplement: Supplementary file 3 [file f1000research-7-17614-s0002.tgz › b2a79496-4ada-47c4-9682-a99ce867836d.zip!/Eight Iranian Health Journals/Archives of Rehabilitation_41.docx]

© Archives of Rehabilitation. This work is licensed under CC BY-NC 4.0 (https://creativecommons.org/licenses/by-nc/4.0/).

Volume & Issue: دوره 10 - شماره 3

Title: رابطه گوژپشتی، فرورفتگی قوس کمری و چرخش لگن با افتادگی احشاء لگنی

http://rehabilitationj.uswr.ac.ir/article-1-379-fa.html

هدف: با توجه به شیوع نسبتاً بالای افتادگی یا پرولاپس احشاء لگنی در زنان و لزوم بررسی تأثیر عوامل مستعد کننده این بیماری در جوامع مختلف، این تحقیق با هدف بررسی رابطه تغییرات انحنای ستون فقرات پشتی و کمری و چرخش (تیلت) لگن با پرولاپس احشاء لگنی انجام شد. روش بررسی: در این مطالعه مورد-شاهدی، ۱۵نفر از زنان مبتلا به پرولاپس احشاء لگنی از طریق نمونه‌گیری ساده و در دسترس و با توجه به معیارهای لحاظ شده، از بین بیماران مراجعه‌کننده به بیمارستان‌های اکبرآبادی و امام‌خمینی به‌عنوان گروه مورد و ۱۵ نفر از مراجعین همان بیمارستان‌ها که درجه پرولاپس آنها صفر و یا یک بود، بر اساس همتاسازی با گروه مورد، به‌عنوان گروه شاهد انتخاب و وضعیت پرولاپس (با استفاده از سیستم اندازه‌گیری پرولاپس احشاء لگنی)، انحناهای ستون فقرات پشتی و کمری (با استفاده از خط کش قابل انعطاف) و تیلت لگن (با استفاده از شیب سنج لگنی) آنها مورد بررسی و مقایسه قرار گرفت. داده‌های حاصل با استفاده از آزمون‌های آماری کولموگروف-اسمیرنوف، تی مستقل و رگرسیون لجستیک تجزیه و تحلیل شد. یافته‌‌ها: بین دو گروه اختلاف معناداری از لحاظ میزان کایفوز پشتی، (۰/۲۶=P)، لوردوز کمری (۰/۵۲=P) و تیلت لگن (۰/۴۱=P) وجود نداشت. نتیجه‌گیری: گرچه مطابق نتایج این مطالعه، ارتباط معناداری بین میزان کایفوز پشتی، لوردوز کمری و تیلت لگن با بروز پرولاپس احشای لگنی یافت نشد، اما همسویی جهت تغییرات فاکتورهای مورد نظر با مفروضات مطالعه، وجود رابطه بین افزایش کایفوز و کاهش لوردوز با بروز پرولاپس احشاء لگنی را در یک جامعه بزرگتر آماری، همچنان محتمل باقی نگاه می دارد.

Title: مقایسه برخی توانایی‌های کاربرد شناختی کودکان فارسی زبان کم‌شنوای شدید 4 تا 6 ساله با همسالان شنوا

http://rehabilitationj.uswr.ac.ir/article-1-378-fa.html

هدف: کاربردشناسی زبان عبارت از مطالعه کنشهای گفتاری و جنبه های اجتماعی تعاملات ارتباطی در بافت زبان است. کودکان کم‌شنوا، به دلیل محروم بودن از حس شنوایی که نقش مهمی در رشد مهارت‌های زبانی دارد، در تمام سطوح زبان و از جمله کاربردشناسی با مشکلاتی مواجهند. هدف از این پژوهش، مقایسه برخی توانایی‌های کاربردشناختی شامل پیگیری موضوع، نوبت‌گیری و اصلاح مکالمه در کودکان فارسی زبان کم‌شنوای شدید 4 تا 6 ساله با همسالان شنوا می‌باشد. روش بررسی: در این پژوهش مقطعی مورد-شاهدی، 21 کودک شنوا به روش تصادفی ساده از مهدکودکهای عادی و 21 کودک کم‌شنوای شدید به روش نمونه گیری دردسترس از مراکز توانبخشی و کلینیکهای گفتاردرمانی تهران و کرج انتخاب شده و مهارت‌های کاربردشناسی آنها با استفاده از تکالیف محقق‌ساخته در خصوص پیگیری موضوع، نوبت‌گیری و اصلاح مکالمه مورد بررسی قرار گرفت. روایی محتوایی تکالیف، توسط یک زبان شناس و دو آسیب شناس گفتار و زبان و پایایی تمامی آنها به روش آزمون-بازآزمون و ضریب همبستگی 0/70 تا 0/95 مورد تأیید قرار گرفت. لازم به‌ذکر است ابتدا خرده آزمونهای هوش غیرکلامی از آزمودنیها به عمل آمد و در صورتی که نمره هوش آزمودنی در محدوده طبیعی به دست می آمد، تکالیف کاربرد شناختی در اختیار او قرار می گرفت. داده‌های حاصل با استفاده از آزمونهای مجذور خی، تی مستقل و مان ویتنی مورد تجزیه و تحلیل قرار گرفت. یافته‌ها: میانگین مدت‌زمان پیگیری موضوع (P<0/001)، نمرات نوبت‌گیری (P<0/001)، اصلاح مکالمه از نوع پاسخ به درخواست برای رفع ابهام (P=0/003) و اصلاح مکالمه از نوع درخواست برای رفع ابهام (P=0/002) بین دو گروه کم‌شنوا و شنوا اختلاف معناداری داشت. نتیجه‌گیری: کودکان کم‌شنوا در به‌کارگیری مهارت‌های کاربردشناسی ضعیف‌تر از کودکان شنوا عمل می‌کنند.

Title: مقایسه الگوی فعالیت عضلانی کمربند شانه‌ای در فعالیت‌های عملکردی انتخابی بین بیماران مبتلا به سندرم‌ گیرافتادگی شانه و افراد سالم

http://rehabilitationj.uswr.ac.ir/article-1-377-fa.html

هدف: هدف این تحقیق مقایسه الگوی فعالیت عضلانی کمربند شانه‌ای در بیماران دچار سندرم گیرافتادگی شانه و افراد سالم در الگوهای حرکتی عملکردی می‌باشد. روش بررسی: در این مطالعه مقایسه‌ای مورد-شاهدی، ۱۵ بیمار مبتلا به سندرم گیرافتادگی شانه به‌صورت ساده و در دسترس از بین مراجعین کلینیک ارتوپدی اوستا و ۱۵ فرد سالم از طریق همتاسازی با بیماران انتخاب و با استفاده از ثبت الکترومیوگرافی سطحی از عضلات سینه‌ای بزرگ، ذوزنقه‌ای فوقانی و میانی، تحتِ خاری و سه بخش عضله سه‌گوش (دلتوئید)، فعالیت الکتریکی و وارد عمل شدن عضلات مذکور در حین فعالیت‌های عملکردی الگوهای حرکتی زنجیره‌ باز با و بدون اعمال مقاومت خارجی و الگوهای حرکتی زنجیره بسته توأم با مقاومت محوری در دو گروه مورد ارزیابی و مقایسه قرار گرفت. برای تحلیل داده ها از آزمون تی مستقل استفاده شد. یافته‌ها: الگوهای زمانی متفاوتی از توالی وارد عمل شدن عضلات در هر یک از دو گروه سالم و بیمار حین فعالیت‌های عملکردی مذکور وجود داشت. به این ترتیب که زمان وارد عمل شدن هر یک از عضلات ذوزنقه‌ای فوقانی، تحتِ خاری و سه بخش عضله سه‌گوش بین دو گروه اختلاف معنا‌دار داشت (P<0/05). نتیجه‌گیری: تغییرات در الگوی فعالیت عضلانی رابطه تنگاتنگی با الگوی حرکتی مورد ارزیابی دارد که این رابطه ناشی از راستای انجام حرکت و میزان فشار بر روی فضای آناتومیک هدف است. الگوی حرکتی دی ۲ اکستنشن کمترین تغییرات و الگوی حرکتی تری‌پاد بیشترین تغییرات را در حوزه زمان از خود نشان دادند.

Title: تأثیر برنامه خودمراقبتی مبتنی‌بر الگوی اورم بر میزان خستگی و فعالیت‌های روزانه زندگی بیماران مبتلا به مولتیپل اسکلروزیس

http://rehabilitationj.uswr.ac.ir/article-1-376-fa.html

هدف: خستگی یکی از زجر‏آورترین علائم بیماری مولتیپل اسکلروزیس است که با کاهش کلّی فعالیت‏های مراقبت از خود باعث محدودیت ایفای نقش شده و توانایی فرد را برای انجام فعالیت‏های روزمره محدود می‏کند. این مطالعه با هدف تعیین تأثیر برنامه خودمراقبتی مبتنی بر الگوی اورم بر خستگی و فعالیتهای روزانه زندگی بیماران مولتیپل اسکلروزیس انجام گرفت. روش بررسی: این مطالعه تجربی با طراحی کارآزمایی بالینی و با حضور 70 بیمار مبتلا به مولتیپل اسکلروزیس که به‌صورت تصادفی از بین بیماران واجد شرایط انجمن ام.اس.انتخاب شده و به روش تصادفی‌تعادلی به دو گروه آزمون و شاهد تخصیص یافتند انجام شد. برای گروه آزمون 8 جلسه برنامه آموزشی بر اساس نیازهای آموزشی بیماران و مبتنی بر الگوی اورم اجرا گردید. این برنامه در طول سه ماه توسط بیماران به‌کار گرفته شد و با استفاده از چک لیستهای خودگزارش‌دهی میزان به‌کارگیری برنامه ها پیگیری و اندازه گیری گردید. خستگی و فعالیت‏های روزمره بیماران دو گروه قبل و سه ماه بعد از اجرای برنامه با استفاده از مقیاس خستگی پایپر و مقیاس بارتل مورد ارزیابی قرار گرفت. داده ها با استفاده از آزمون های آماری مجذور خی، تی‌ زوجی و مستقل و آنالیز واریانس مورد تجزیه و تحلیل قرار گرفت. یافته‌ها: بین دو گروه اختلاف معناداری در میزان خستگی (۰/۴۸=P) و فعالیت‏های روزمره (۰/۲۴=P) قبل از مداخله وجود نداشت، درحالی‌که بعد از مداخله تفاوت دو گروه معنادار شد (۰/۰۰۱>P). میزان خستگی و فعالیت‏های روزمره بیماران گروه آزمون بعد از مداخله نسبت به قبل از آن کاهش معناداری یافت (۰/۰۰۱>P)، درحالی‌که میزان خستگی (۰/۰۸۶>P) و فعالیت‏های روزمره (۰/۳۳>P) گروه شاهد بعد از سه ماه تفاوت معناداری نداشت. نتیجه‌گیری: به‌کارگیری برنامه خودمراقبتی مبتنی بر الگوی اورم و بر اساس نیازهای آموزشی به بیمار به‌عنوان یک مداخله پرستاری غیرتهاجمی و کم‌هزینه بر میزان خستگی و فعالیت‏های روزمره بیماران مولتیپل اسکلروزیس مؤثر می باشد.

Title: بررسی تأثیر آموزش نحوه مراقبت از کودکان مبتلا به فلج‌مغزی بر کیفیت‌ زندگی مراقبین خانوادگی آنها

http://rehabilitationj.uswr.ac.ir/article-1-375-fa.html

هدف: نظر به اینکه وجود فرزند مبتلا به فلج‌مغزی زندگی خانواده و به‌ویژه مراقب خانوادگی کودک را متأثر می سازد، مطالعه حاضر با هدف تعیین تأثیر آموزش نحوه مراقبت از کودکان مبتلا به فلج‌مغزی بر کیفیت زندگی مراقبین خانوادگی این کودکان انجام شده است. روش بررسی: در این مطالعه مداخله‌ای و شبه‌تجربی، ۲۵ نفر از مراقبین اصلی خانواده‌‌های دارای کودک مبتلا به فلج‌مغزی اسپاستیک تحت پوشش بهزیستی شهرستان بابلسر که حداقل سواد خواندن و نوشتن داشتند، به‌روش تمام‌شمار و با رعایت معیارهای لازم انتخاب و مورد آموزش قرار گرفتند. قبل و سه‌ماه پس از آموزش، میزان مهارت مراقبین در مراقبت از کودک مبتلا به فلج‌مغزی بر اساس فرم جمع‌آوری اطلاعات نحوه مراقبت از کودکان فلج‌مغزی و کیفیت‌زندگی مراقبین با پرسشنامه فرم کوتاه بررسی وضعیت سلامت (SF36) مورد سنجش قرار گرفت. آموزش به‌صورت عملی و با حضور مراقب و کودک مبتلا در طی 6 تا ۸ جلسه 30 دقیقه‌ای انجام پذیرفت. داده‌های حاصل با استفاده از آزمون تی‌زوجی و آزمون ناپارامتری ویلکاکسون تحلیل شد. یافته‌ها: نمرات کیفیت‌زندگی مراقبین 3 ماه پس از پایان آموزش نسبت به قبل از دریافت آموزش در تمامی 8 قلمرو، 2 حیطه کلی سلامت‌ذهنی و سلامت‌جسمانی و کیفیت‌زندگی کل به میزان معناداری افزایش یافت (P<0/001). از میان قلمروهای هشت‌گانه کیفیت‌زندگی، کمترین میانگین افزایش در قلمرو سلامت‌ذهنی (54/13±36/11) و بیشترین آن در قلمرو نقش‌عاطفی (24/78±66/50) مشاهده شد. میزان این تغییر در نمره کلی کیفیت‌زندگی (83/15±18/23) بود. نتیجه‌گیری: با توجه به ارتقاء کیفیت‌زندگی والدین به‌دنبال گذراندن دوره آموزشی درباره نحوه صحیح مراقبت از کودکان مبتلا به فلج‌مغزی، اهمیت آموزش والدین بیش از گذشته معلوم شده و به نظر می‌رسد در ارزیابی اثربخشی برنامه‌های توانبخشی کودکان، می‌بایست آثاری که این برنامه ها بر سایر اعضاء خانواده دارد نیز همواره مورد توجه قرار گیرد.

Title: بررسی تأثیر فعالیت‌های حرکتی درشت بر فرایند توجه دانش‌آموزان پسر مبتلا به نشانگان داون

http://rehabilitationj.uswr.ac.ir/article-1-374-fa.html

هدف: یکی از موارد مهم و مورد توجه در کودکان، توانایی و یا ناتوانی در انجام فعالیت‌های حرکتی است که در رشد عملکردهای شناختی و رفتارهای اجتماعی کودک تأثیر می‌گذارد. هدف این مطالعه بررسی تأثیر فعالیت‌های حرکتی درشت بر فرایند توجه (توجه پایدار، تغییر توجه) دانش‌آموزان پسر نشانگان داون ۸ تا ۱۲ ساله می‌باشد. روش بررسی: در این مطالعه مداخله‌ای و تجربی، 26 دانش‌آموز مبتلا به نشانگان داون به‌صورت هدفمند از 4 مدرسه کودکان استثنایی شهید بهشتی، احیا، پیدایش و پیروزی انتخاب شده و با آزمون‌های توجه (توجه پایدار و تغییر توجه) و مقیاس رشدی ازرتسکی مورد ارزیابی اولیه قرار گرفتند. سپس نمونه‌ها به‌صورت تصادفی‌تعادلی به دو گروه مداخله (13 نفر) و کنترل (13 نفر) تخصیص یافتند. برای گروه مداخله، مداخلات با تأکید بر فعالیت‌های حرکتی درشت به مدت 8 هفته (هفته‌ای 2 بار) انجام گرفت. بعد از اتمام مداخلات، دو گروه مجدداً تحت ارزیابی قرار گرفته و داده‌های حاصل با استفاده از آزمون تی زوجی و تی مستقل مورد تجزیه‌و‌تحلیل آماری قرار گرفت. یافته‌ها: مقایسه نمرات آزمون‌های توجه قبل و بعد از مداخله در گروه کنترل (توجه پایدار P=0/654 و تغییر توجه P=0/163) و مقیاس رشدی ازرتسکی (P=0/377)، حاکی از عدم اختلاف معنادار بود، اما در گروه مداخله تأثیر معنادار مداخلات دیده شد (P<0/001). همچنین در نمره مربوط به کاهش انقطاع از تکلیف، بین میانگین سطح نمرات هر دو گروه مداخله (P<0/001) و کنترل (P<0/001) اختلاف معناداری وجود داشت. نتیجه‌گیری: تقویت فعالیت‌های حرکتی درشت بر فرایند توجه (توجه پایدار و تغییر توجه) دانش‌آموزان نشانگان داون به‌طور معناداری مؤثر است.

Title: تأثیر آموزش گروهی والدین کودکان 4 تا 10 ساله مبتلا به اختلال نقصان توجه-بیش‌فعالی بر اختلالات رفتاری فرزندانشان

http://rehabilitationj.uswr.ac.ir/article-1-373-fa.html

هدف: اختلال نقص توجه – بیش فعالی شایعترین اختلال عصبی رفتاری دوران کودکی می باشد که برای بهبود تعاملات اجتماعی و عاطفی ایشان به مداخلات روانی اجتماعی نیاز است و یکی از تأثیرگذارترین این برنامه ها آموزش گروهی والدین می باشد. هدف این مطالعه بررسی تأثیر برنامه آموزش گروهی والدین بر میزان اختلالات رفتاری کودکان دچار نقص توجه- بیش‌فعالی می باشد. روش بررسی: در این مطالعه شبه تجربی 54 کودک 4 تا 10 ساله مبتلا به اختلال نقص توجه- بیش‌فعالی، از بین مراجعه‌کنندگان به 7 مرکز کاردرمانی سطح شهر تهران به‌روش ساده و در دسترس انتخاب و به طور تصادفی‌تعادلی به دو گروه شاهد و مداخله اختصاص یافتند. علاوه بر برنامه های کاردرمانی و دارودرمانی مشترک در هر دو گروه، به مادران کودکان گروه مداخله، به مدت 8 جلسه دو ساعته و به‌صورت هفتگی برنامه آموزش گروهی والدین طبق دستورالعمل فرزندپروری مثبت ساندرز (2005) ارائه شد. ابزار گرد آوری داده ها شامل پرسشنامه اطلاعات دموگرافیک و پرسشنامه 48 سئوالی مقیاس رده‌بندی والدین کانرز بود. داده‌های حاصل به وسیله آزمون های آماری مجذور خی، تی زوجی، تی مستقل و آنالیز کوواریانس تحلیل شد. یافته‌ها: علی رغم بهبود معنادار هر دو گروه در ارزیابی درون‌‌گروهی قبل و بعد، میزان کاهش نمرات خرده مقیاس های رفتاری سلوک (P<0/001)، اضطراب (P<0/001)، شاخص بیش‌فعالی (P=0/016) و نمره کل پرسشنامه (P<0/001) در گروه مداخله نسبت به گروه شاهد معنادار بود. در حالی که در خرده مقیاس های یادگیری (P=0/052)، تکانشی بیش فعالی (P=0/267) و مشکلات روان‌تنی (P=0/526) تغییر معناداری ایجاد نشد. نتیجه‌گیری: آموزش گروهی والدین کودکان مبتلا به اختلال نقص توجه-بیش‌فعالی در کاهش میزان اختلالات رفتاری و اضطرابی ایشان تأثیر گذار است و این مداخله می تواند در کنار برنامه های دارودرمانی و کاردرمانی سودمند واقع شود.

Title: زمان آغاز واکداری در همخوان‌های زبان آذری

http://rehabilitationj.uswr.ac.ir/article-1-372-fa.html

هدف: زمان آغاز واکداری سرنخی جهت افتراق همخوان های بندشی واکدار و بی واک بوده و می تواند جهت توصیف یا طبقه بندی طیفی از اختلالات رشدی، عصبی- حرکتی و یا زبانی مورد استفاده قرار گیرد. هدف این مطالعه تعیین مقادیر هنجار زمان آغاز واکداری در زبان آذری (لهجه تبریز) می‌باشد. روش بررسی: در این مطالعه توصیفی- تحلیلی30 گوینده آذری زبان که به‌صورت ساده و در دسترس انتخاب شده بودند، پس از شنیدن 46 تک کلمه آذری که با همخوان های بندشی آغاز می شد، در دو مرحله آنها را تکرار کرده و صدایشان ضبط شد. سپس توسط نرم افزار پرات، مقادیر میانگین برای زمان آغاز واکداری با تحلیل طیف صوتی و موج شکل صوتی بر حسب میلی ثانیه محاسبه گردید. همچنین تفاوت های بین جنسی و نیز تأثیر واکه ها بر زمان آغاز واکداری بررسی و داده‌های حاصل با استفاده از آزمون تحلیل واریانس یکطرفه و تی مستقل ارزیابی شد. یافته‌ها: بین مردان و زنان آذری زبان تفاوت معناداری در زمان آغاز واکداری وجود نداشت (P<0/05) و محل تولید همخوان (0/001>P) با زمان آغاز واکداری وجود داشت. میانگین مقادیر زمان آغاز واکداری برای واج های/g/،/t/،/p/،/d/،/b/و /k/و دو واجگونه [ɟ] و [c] به ترتیب برابر با 26/25، 87/09، 13/35، 86/88، 10/64، 100/62، 63/18 و 131/19 میلی ثانیه بود. نتیجه‌گیری: جنسیت کاربران زبان آذری تأثیری بر زمان آغاز واکداری همخوان های بندشی آنها ندارد، اما مشابه سایر زبان ها، واکه های بسته و پسین و محل تولید پسین تر سبب افزایش مقدار آن می گردد. همچنین همخوان های بی واک از نوع دمشی بوده و همخوان های واکدار دارای مقادیر زمان آغاز واکداری مثبت می باشد.

Title: تأثیر بازی‌های آموزشی بر میزان یادگیری برخی از مفاهیم ریاضی در دانش‌آموزان پسر کم‌توان ذهنی آموزش‌پذیر

http://rehabilitationj.uswr.ac.ir/article-1-371-fa.html

هدف: هدف این پژوهش تعیین تأثیر بازی‌های آموزشی بر میزان یادگیری برخی از مفاهیم ریاضی در دانش‌آموزان پسر کم‌توان ذهنی آموزش‌پذیر است. روش بررسی: در این پژوهش شبه تجربی، به روش نمونه‌گیری در دسترس، دو مدرسه کودکان کم‌توان ذهنی انتخاب شده و تمام دانش‌آموزان پایه اول ابتدایی (قسمت اول) این دو مدرسه به‌صورت تصادفی در دو گروه آزمایش و کنترل جایگزین گردیدند (هر گروه 8 نفر). سپس گروه آزمایش مفاهیم ریاضی را از طریق بازی‌های آموزشی و گروه کنترل به روش جاری تدریس آموزش دیدند. ابزارهای پژوهش شامل4 بازی آموزشی محقق‌ساخته، 4 خرده آزمون محقق‌ساخته و یک پس آزمون محقق‌ساخته بود. داده‌های حاصل با استفاده از آزمون «یو من‌ویتنی» تحلیل شد. یافته‌ها: تفاوت میانگین نمرات دو گروه آزمایش و کنترل در کل مفاهیم ریاضی معنادار بود (P=0/025). همچنین این مداخله باعث افزایش میانگین نمرات گروه آزمایش در هر یک از مفاهیم ریاضی نسبت به گروه کنترل شد، اما این اختلاف معنادار نبود. نتیجه‌گیری: بازی‌های آموزشی می‌تواند موجب ارتقاء میزان یادگیری مفاهیم ریاضی در دانش‌آموزان کم‌توان ذهنی شود.
